# The COVID-19 experience and the necropolitical space of end-of-life care in residential care facilities in Quebec: A case study

**DOI:** 10.1177/26323524251407703

**Published:** 2026-02-13

**Authors:** Andréanne Robitaille, Johanne Collin, Pierre-Marie David

**Affiliations:** 1 Faculté de pharmacie, Université de Montréal, QC, Canada; 2 Institute for Circumpolar Health Research, Yellowknife, NT, Canada

**Keywords:** necropolitical space, home care, end of life, COVID-19, residential care facilities, Québec

## Abstract

**Background:**

The COVID-19 pandemic highlighted the need for innovative approaches to healthcare delivery, particularly for older adults with frailty in crowded settings. In Québec, Canada, the shortage of hospital beds during the pandemic exposed critical gaps in the capacity to provide appropriate end-of-life care for affected residents of residential care facilities (RCFs).

**Aim:**

This study aimed to examine the implementation of the COVID-19 Intensive Home Care Team (IHCT) and how it transformed the physical and social environment of RCFs to enable end-of-life care and “dying at home” in an unprecedented time.

**Design:**

A qualitative case study design was used. Between September 2020 and January 2021, qualitative data were collected through 30 in-depth interviews with front-line workers of the IHCT, healthcare managers, and academics in the home care sector. Thematic analysis reveals three interrelated themes: the ambiguities of caring spaces, the centrality of medication management, and the reconfiguration of RCFs through new spatial, technological, and relational arrangements that enabled “dying at home” during the COVID-19 pandemic.

**Results:**

Findings revealed that the IHCT initiative reshaped RCFs into a necropolitical space, showing the complex interplay between space, care practices, and societal values toward older adults with frailty. The transformation enabled a form of compassionate end-of-life care within familiar settings as well as exposed underlying societal hierarchies that determine whose lives, and deaths, are valued.

**Conclusion:**

The IHCT model illustrates how emergency health interventions can reconfigure care spaces and challenge conventional boundaries between home, private, and public care systems. However, it also underscores the ethical and political dimensions of care for frail older adults in crisis contexts.

## Introduction

The physical place where one dies represents a political, moral, and emotional space that reflects societal values and the complex dynamics shaping end-of-life experiences.^1–3^ The preference for home as the place of death is often seen as “common sense,” but this view carries significant sociopolitical and equity implications. Public policies reinforce an idealized vision of dying at home, promoting support initiatives through the narrative of choice, preference, care, and need.^4^ However, public preferences and decision-making regarding end-of-life care become increasingly complex when they intersect with material realities and inequalities.^4^

An aging population raises important questions for society, particularly about end-of-life care in facilities for older adults, such as residential care facilities (RCFs).^3,5^ RCFs, managed by private companies, are designed primarily for adults aged 65 and older who either live independently in apartments or require assistance because of physical or cognitive limitations. Such residents rent private apartments while receiving specific care services provided by the facility, and they may also access public healthcare services. There is an interplay between private and public care systems, which creates complexity for access to end-of-life support.^6^ To our knowledge, few studies have specifically examined end-of-life care in relation to space and place within these settings.^5,7^

The COVID-19 pandemic has significantly influenced societal attitudes toward death and highlighted the importance of end-of-life care.^8,9^ The vulnerabilities of older adults living in long-term care facilities and those receiving home care were starkly revealed worldwide, as the pandemic disproportionately affected residents of aged care settings.^10–14^ Frail older adults who contracted COVID-19 were isolated and left without adequate medical attention, which resulted in many dying alone.^11,14^ Home care workers experienced difficulty accessing personal protective equipment, training, and adequate support, also exposing caregivers and older adults to increased risks.^14,15^

The pandemic demonstrated the need for systemic change to address neglect and inadequacies in the care of older adults with frailty, prompting a re-evaluation of health policies, resource allocation, and the overall treatment of the aging population.^16–18^ The crisis brought to light the necropolitical rationale guiding life-and-death decisions, revealing the state’s authority in determining survival, particularly in aged care settings, such as hospital triage protocols that determined who received access to critical resources.^19^ The term “necropolitical” refers to power relations that govern life and death, disproportionately affecting marginalized communities.^20^

During the first wave, Québec (Canada) had the highest incidence and mortality rates among Canadian provinces, positioning it as a “textbook case” for analyzing the preparedness of the health and social services system in managing health crises.^21^ The majority of COVID-19-related deaths were observed in individuals aged 70 and above, and represented 92% of total fatalities. Within this age group, 64.3% of deaths took place in long-term care facilities, while 16.5% occurred in RCFs.^22^ In Québec, 18.5% of people (compared to 6.1% in Canada) aged 75 and over live in RCFs and require less than 1.5 h of daily care.^23^ Yet, the degree of autonomy among residents in Québec’s RCFs is significantly influenced by the shortage of long-term care facilities and limited funding for home care within the healthcare system.^24^ These facilities are not just residential spaces but politically charged environments that reflect public policy priorities and decisions.^25^ The COVID-19 pandemic exposed the consequences of past and present policy choices through the high mortality rates observed and the lived experiences of residents and staff.

This study examined how RCFs were transformed to facilitate healthcare-assisted death at home under exceptional circumstances. We aimed to understand how care practices transformed the physical and social environments of RCFs to meet, or fail to meet, the needs associated with “dying at home” during an unprecedented situation. We identified complexities that highlight the challenges and ambiguities of this process.

### Dying at home

Palliative care delivered in the home is considered a more humane approach to end-of-life care, extending beyond purely biomedical concerns.^3,4,26^ Accepting a patient’s decision to die at home is viewed as a means of respecting their dignity and autonomy, while also aligning with the need for healthcare systems to cut costs as they face an aging population.^2,27^ By promoting this option through public policy, healthcare systems can align with patients’ expectations and optimize resource utilization.^4,28^

However, dying at home is not always ideal^29,30^ or universally preferred.^31^ The diversity of housing situations, and what someone calls “home,” has significant implications for end-of-life care, as the place of residence influences who provides care and how it is delivered.^2^ Patients and families prioritize well-being and social support at the end of life, regardless of the location.^32^ Dying at home presents challenges, including frequent intrusions by healthcare professionals (HCPs) and the medicalization of domestic space,^33^ and places a considerable burden on family caregivers.^32^ The home environment itself can be a therapeutic or obstructive environment, depending on the available support and resources.

### The necropolitical space

The concept of “place” emphasizes the importance of geographical and social location, whereas “space” encompasses more abstract and symbolic dimensions of experience.^1^ Spaces influence health and well-being because they shape the social construct and dynamic interactions between the environment, people, and care practices.^34^ State and citizen responsibilities for care are expressed through these spatial arrangements.^3^ Home care operates within a space that highlights a series of interrelated distinctions between the public and the private, and implicates power dynamics in care relationships.^6^ Providing quality palliative and end-of-life care at home requires a relationship-centered approach, which was significantly disrupted during the COVID-19 pandemic.^14^

RCFs were not originally intended as places where people would spend their final days. They evolved into end-of-life settings due to factors such as an aging population, healthcare policies, and changes in family structures, which influenced this spatial transformation.^3^ In this study, we argue that under the COVID-19 conditions, RCFs functioned as necropolitical spaces. As microcosms of broader societal issues, they revealed critical concerns about equity, accessibility, and quality of care for older adults with frailty. The idea of “dying at home,” when applied to these settings, presented significant challenges. Although residents were expected to retain some autonomy, their choices regarding end-of-life care became severely restricted during the pandemic. By examining these changes through the lens of necropolitical space,^19^ we argue that during the pandemic, RCFs adopted a more hospital-centric model, redefining the concept of “home” as an institutionalized care space, ultimately reframing the way death occurred within these spaces.

## Materials and methods

This qualitative case study^35,36^ was conducted between September 2020 and January 2021, during the second wave of COVID-19 in the province of Québec. We focused on examining end-of-life care practices implemented in RCFs during the initial wave through a specific initiative from the public healthcare system: the COVID Intensive Home Care Teams (COVID-IHCTs). IHCTs provide prompt home visits and short-term follow-up care by physicians and nurses for individuals with frailty facing acute health issues at home.^37^ COVID-IHCTs were implemented in a specific geographic area for a limited period during the critical situation of the pandemic, and to date, only a limited number of IHCTs have been established in Québec. That makes this a particularly relevant case study to examine how RCFs were transformed to facilitate healthcare-assisted deaths under exceptional circumstances. This research is reported in accordance with the Consolidated criteria for reporting qualitative research (COREQ) checklist^38^ (see Supplemental File 1), ensuring transparency and trustworthiness in study design and reporting.

### Study context

In March 2020, following the Government of Québec’s declaration of a health emergency, the Department of Health and Social Services advised that older individuals categorized as “frail” should remain at home if infected with COVID-19. This measure aimed to alleviate pressure on hospitals and the healthcare system, which were already strained by the surge in patients. The provincial healthcare system’s inadequacy in managing the crisis became evident by the shortages in protective gear and medical resources.^21^ In response, several integrated health and social services centers (IHSSCs) formed IHCTs to support older adults with COVID-19 receiving care at home.^39^ An IHSSC is a public institution responsible for coordinating and delivering a continuum of health, social, and community services to a defined population within a specific region.

During the initial wave of the pandemic, one IHSSC set up a COVID-IHCT to address the needs of the aging population within its jurisdiction, primarily focusing on RCFs. This region, covering more than 200 km, served by a single hospital responsible for more than 430,000 residents, of whom 17.4% were aged 65 and older.^40^ This IHSSC accounted for 12% of all COVID-19-related deaths during the spring of 2020 and was significantly impacted.^22^ To understand the rise in deaths during the first wave, the IHSSC conducted an internal investigation and subsequently approved our research proposal to study its COVID-IHCT initiative in the summer of 2020. Additionally, this IHSSC was involved in the coroner’s inquiry into Québec’s management of deaths among older adults and vulnerable individuals in residential care during the first wave of COVID-19.^41^ According to internal documents, of the 149 individuals with COVID-19 who received care from the COVID-IHCT team between April 5 and June 5, 2020, 39 died at “home” (at their RCF).

### Participants and sample

Purposive sampling was used to recruit study participants. All HCPs (*n* = 35) directly involved in the first wave of the COVID-IHCT initiative were invited to participate by email. Recruitment was challenging during the second wave of the pandemic, despite repeated reminders, as clinicians faced heightened demand and exhaustion. In addition, experienced system-level managers from the IHSSC and RCFs involved with the COVID-IHCT initiative, as well as academics in the Québec home care sector, each with at least 10 years of experience, were recruited. These three groups were selected to capture diverse perspectives on the COVID-IHCT initiative and to address the fact that the perspectives of HCPs are often overlooked.^42,43^ Participants did not receive any compensation.

A total of 30 participants were included, representing HCPs (*n* = 17), managers and directors (*n* = 8), and academics (*n* = 5). HCPs included family physicians, nurses, nurse practitioners, pharmacists, and a physiotherapist, primarily from IHSSC A, with additional representation from IHSSCs B and C and a private community pharmacy. Managers and directors included leaders from IHSSC A and two RCFs (RCF 1 and RCF 2), holding roles such as nurse manager, director of nursing, and executive director. Academics were affiliated with three universities and specialized in sociology, nursing, geriatrics, and gerontology. Collectively, participants provided a multisectoral and multidisciplinary perspective on the COVID-IHCT initiative.

Table 1 provides a list of participants. To ensure confidentiality, particularly given the coroners’ inquiry, participants are identified only by role-based codes, with details such as gender, age, and exact location removed.

**Table 1.** Data sheet of participants.

Role	Institution	Participant code
Interviews with healthcare professionals COVID-IHCT		
Family physician	IHSSC A	HCP2
Family physician	IHSSC A	HCP9
Family physician	IHSSC A	HCP12
Family physician	IHSSC A	HCP13
Family physician	IHSSC B	HCP4
Family physician	IHSSC B	HCP5
Family physician	IHSSC C	HCP6
Nurse	IHSSC A	HCP3
Nurse	IHSSC A	HCP8
Nurse	IHSSC A	HCP10
Nurse	IHSSC A	HCP11
Nurse	IHSSC A	HCP17
Nurse practitioner	IHSSC A	HCP7
Nurse practitioner	IHSSC A	HCP15
Pharmacist	Private Community Pharmacy	HCP1
Pharmacist	IHSSC A	HCP14
Physiotherapist	IHSSC A	HCP16
Interviews with managers and directors		
Director of Continuum of Care	IHSSC A	M1
Director of Nursing	RCF 1	M7
Executive Director and Nurse	RCF 2	M8
Nurse – Manager	IHSSC A	M2
Nurse – Manager	IHSSC A	M3
Nurse – Manager	IHSSC A	M5
Owner and Executive Director	RCF 1	M6
Respiratory Therapist – Manager	IHSSC A	M4
Interviews with academics		
Academic – Geriatric medicine	University 2	R2
Academic – Geriatric medicine	University 2	R5
Academic – Gerontology	University 1	R3
Academic – Nursing	University 3	R4
Academic – Sociology	University 1	R1

COVID-IHCT: COVID Intensive Home Care Team; HCP: healthcare professional; IHSSC: integrated health and social services center; RCFs: residential care facilities.

### Data collection and analysis

The first author conducted and recorded virtual in-depth interviews^44,45^ entirely in French using various platforms such as Zoom, Teams, or telephone. Sessions lasted from 30 to 160 min, with an average of 90 min per session. Open-ended questions were used to explore participants’ experiences with the COVID-IHCT, and aimed to avoid oversimplified positive or negative narratives. Comparative analysis among participant answers facilitated deeper insight and identified emerging themes. After 24 interviews, no new themes emerged. However, 6 additional interviews were completed to ensure the inclusion of all 30 interested participants.

During the interviews, several participants expressed that it was beneficial to share their stories, often accompanied by mixed emotions and self-doubt, and appreciated the opportunity for such expression. Participants were offered emotional support resources; however, all reported feeling well supported during the interviews and did not require additional assistance. Given the ongoing coroners’ inquiry at the time, many participants also requested explicit assurances of confidentiality and anonymity. To enhance the trustworthiness of the study, standardized interview guides were used for each group, participants were anonymized, and data were triangulated across multiple HCP categories and participant groups during analysis.

Audio recordings were transcribed using NVivo software (Lumivero) and carefully verified for accuracy, with all personal identifiers removed to preserve anonymity. Thematic analysis^46^ followed a two-stage process: initial open coding to develop a preliminary code list using Dedoose software (Sociocultural Research Consultants, LLC), followed by thematic coding to identify commonalities and differences across responses. Memos and concept maps were maintained throughout both data collection and analysis stages. The first author, who has 20 years of nursing experience, had no prior affiliation with the IHSSC studied. This case study formed part of her doctoral research in social sciences. Reflexivity was maintained throughout the analysis, with the first author’s professional background acknowledged as a potential influence on data interpretation. Themes and associated verbatim excerpts were validated collaboratively with two coauthors through multiple sessions and document reviews conducted between 2021 and 2024, incorporating materials such as press analyses, internal documents, and the Palli-Science website referenced by participants. The participants were invited to provide feedback on preliminary findings through a virtual session, allowing them to comment on emerging themes. Quotations from interviews were translated into English for inclusion in this article.

## Results

During the first wave, the COVID-IHCT initiative played a critical role in allowing residents to die at RCFs. The team was credited with preventing hospitalization for 121 individuals during that period, which many participants considered a success highlighted in other studies reporting on strategies to reduce hospital overload.^47^ According to the participants, without the COVID-IHCT intervention, individuals with COVID-19 symptoms who were supported at home might have experienced greater suffering or worse conditions before death, as hospitals were prioritized for less frail patients. The analysis highlighted challenges of access to care, particularly in RCFs, underscoring the need for rapid intervention to avoid the type of critical situations seen in long-term care during COVID-19.^48^ In addition, the participants’ narratives occasionally utilized military terminology to convey a sense of being in a state of “war.”^19^

### Ambiguities of caring spaces

#### The (im)mobility of the COVID-IHCT

The COVID-IHCT operated as a mobile unit, with HCPs “deployed” directly to RCFs using their own vehicles. Working in teams of two, or sometimes individually when there was an acute shortage of staff, the team covered large areas that were divided into two distinct geographical zones. The participants noted that despite the logistical challenges, concentrating pandemic outbreak care units in some RCFs “streamlined operations,” reduced travel between different homes, and saved time. This exceptional situation required RCFs to quickly transform into mini-hospitals focused on palliative care due to the expected increase in deaths over a short period.^49^

#### The (im)mobility of the patient

A critical factor in determining whether a patient received care at home by the COVID-IHCT or was transferred to the hospital revolved around the patient’s desired level of medical intervention. In contrast to a Do Not Resuscitate (DNR) order, which specifically addresses the refusal of resuscitation efforts in the event of cardiac or respiratory arrest, the level of care refers to the overall spectrum of medical and supportive services tailored to the patient’s preferences. In Québec, there are four levels: Level 1 refers to high-intensity care, Level 2 to intermediate care with some medical support, Level 3 to low-intensity care with minimal medical intervention, and Level 4 refers to comfort care.^50,51^ Managing care at home created significant ambiguity and complex dilemmas for families and the team, especially when patients were unable to give consent because they were too ill. When the patient was unable to consent to care, their appointed substitute decision-maker made the decision.

Despite clear guidelines from the Department of Health advising patients with Level 3 or 4 to remain at their RCF, working with families to make decisions in practice about the health condition of their loved one was complicated and challenging. As a participant pointed out, while the criteria for admission to the COVID-IHCT may seem straightforward, the actual situation was often more complex and ambiguous. Despite the diverse criteria in place, patients often found themselves not fitting neatly into any category:

In the end, we can have a diversity of criteria, but the patient never fits into the right boxes. Technically I could admit patients, but it quickly becomes a factor of exclusion (pause and laughs). (Nurse manager – COVID-IHCT, M5)

Individuals experiencing COVID-19 symptoms with goals for care Level 1 or 2 were often promptly directed to the hospital if there was a likelihood of requiring intubation or respiratory support in an isolation setting to mitigate contamination risks. Yet, families frequently found themselves grappling with remotely determining their loved one’s goal of care, often expressing a strong preference for end-of-life care to be provided at home.

Most of the time, the families didn’t want to transfer them to hospital. They said, “Look, my mother was fine here. If you’re able to give her this kind of care, I’d rather she stayed here.” It wasn’t just us saying we didn’t want to overload the hospital. It was clear that if a family absolutely wanted a transfer there (we would consider it). But most of the time, they didn’t want to. Because there was a big, big job. A job, a race against time. (Family doctor – COVID-IHCT, HCP 13)

Conversely, some patients with care goal Level 3 or 4 and severe disconfort would have benefited from more HCP available and intensified care available at the hospital, but their transfer was not possible according to the guidelines from the Department of Health.

But when patients are in respiratory distress in their own homes, their wishes are not respected. Even if they wanted to die there, they die in respiratory distress, which for me is the worst way to go. Yes, these patients who were very symptomatic or who needed major nursing care, medication, I think they could have been transferred to a red zone in the hospital. (Family doctor – COVID-IHCT, HCP 9)

This circumstance led some families to intentionally choose Level 1 or 2 to ease a future transition if care needs escalated, and others to choose Level 3 or 4 care to honor their loved ones’ preference to die at home. As a result, ambiguity arose from the conflicting dynamics of complying with the ministerial directive to keep patients at home. The decision of distant families, and the team’s recognition of the unmet care needs of some patients, highlighted how care at home extends beyond simply addressing physical bodily needs^3^ and how “institutional space was prioritized via a normative assemblage of acute, curative and longevity logics” (1:4).

#### The public space in a private place

Given the near impossibility of transferring patients to the hospital, the need to transform RCFs into veritable mini-hospitals was discussed by participants. Many of the RCFs where the COVID-IHCT was called upon to intervene lacked protective equipment and personnel. Furthermore, some of the residents were cognitively impaired and continued moving freely around despite having COVID-19 symptoms.

The retirement homes were really unprepared. Sometimes we’d have one case, then the next week we’d have 30 cases in the same retirement home. They didn’t necessarily isolate them. They didn’t test them all. So, it’s certain that if we removed certain superspreaders from the retirement home, we’d have fewer cases in the retirement homes and fewer deaths. (Family doctor – COVID-IHCT, HCP 9)

The COVID-IHCT was itself short of resources across the region to visit the various RCFs. The participants shared that they almost acted as a “scout” team, until the resources organized by the health center took over to offer a minimum of basic care and to implement infection prevention and control measures:

. . . I realized that nothing had been done by the retirement home. There had been no hydration, they were afraid to go into the suspected COVID rooms. So, the patients were simply left on their own for 24–48 hours, 72 hours. . . . Then, finally, it was like a second alarm bell. Then, finally, the personnel from the health centre arrived that evening, and right away, the right equipment arrived, the staff and the service assistants. The service assistants were really very helpful, because they were the ones who made sure that basic care was provided. In fact, they were the ones providing it. (Nurse practitioner – COVID-IHCT, HCP 7)

As reported by the participants, the COVID-IHCT team provided end-of-life care for residents with considerable loss of autonomy awaiting transfer to public long-term care facilities. One participant noted that the COVID-IHCT team assumed responsibility for managing overwhelmed RCFs facing COVID-19 outbreaks among their already vulnerable residents.

It was as if little Martians had arrived, come to destroy and then left again. Because it all happened in three weeks, three and a half weeks. It happened so fast that I called them my little Martians. That’s what I was telling myself in my head, because I was thinking that it didn’t seem right, because I was even frustrated at one point to say, where are the people to come and help us instead of destroying us. Where are the people to come and help the residents stay alive? (Owner and executive director – RCF 1, M6)

This ambiguous situation, the coexistence of private ownership and dependence on public healthcare resources, created palpable tension, as reported in the literature.^19^

#### Territorial medical coverage and medical “desertions.”

Another source of ambiguity frequently highlighted by participants was the historical lack of adequate medical coverage for residents. Access to comprehensive medical care for residents in RCFs was hindered by limited coverage from family doctors, who often do not make home visits. Moreover, if there was a physician affiliated with an RCF, they were often unavailable outside of office hours and not equipped to provide palliative or acute care, exacerbating the challenges in accessing necessary services. Thus, the context of emergency measures during the pandemic removed multiple budgetary and administrative barriers,^52^ favoring the implementation of the COVID-IHCT and its operation, as illustrated by this excerpt:

COVID is particularly deadly in patients over 80, and these patients should be able to benefit from comfort care in their own homes. In times of pandemic, home care physicians are called upon to play the role of firefighters in these retirement homes, where too many seniors fall ill at the same time. Let’s be ready.^49^

From early April to early June 2020, the COVID-IHCT team had extensive territorial medical coverage, particularly in RCFs. A doctor was available 24/7, and the team cared for any patient with or without a family doctor. However, the COVID-IHCT team sometimes found itself in delicate situations, balancing a patient’s regular family doctor versus the territorial medical coverage offered by the COVID-IHCT. Some participants described an intervention in an RCF where the attending physician on duty could not be reached. When they arrived in the middle of the night, several people were ill, and the COVID-IHCT team began end-of-life respiratory distress protocols. However, when the family doctor returned from leave, they disagreed with this approach. On the one hand, the attending physician advocated stopping morphine. On the other hand, for the IHCT team, the reality of patients in respiratory difficulty demanded the administration of morphine to relieve their suffering:

We know that these are patients who are poorly pain relieved and that if we don’t help them, we can’t blame ourselves for letting them suffer. That really hurts, and it was a difficult decision. (Family doctor – COVID-IHCT, HCP 12)

As many residents had no medical coverage, certain situations created a complex medico-legal context in the home. This was particularly true for those unable to consent due to cognitive impairment or severe symptoms, for whom the family and substitute decision-maker placed their trust in the remote COVID-IHCT team.

### Medication management at the heart of space transformation

#### The “baby bottle”: A technical solution

Medication management played a pivotal role in the transformation of the care environment within the RCFs. New care technologies actively reshaped both the physical space and routines of the home.^3^ One key technical solution, referred to as the “baby bottle,” served as a practical adaptation to administer the COVID respiratory distress protocol, an end-of-life protocol modified for COVID-19 symptoms.^53^ These elastomeric pumps^54^ have a small balloon inside that continuously pushes medication by elastic recoil, allowing for the administration of necessary end-of-life medications with minimal patient contact to address staffing shortages and reduce contact with infected patients. This solution not only altered how medications were delivered but also allowed adjustments to room layouts in COVID times, staff workflows of the COVID-IHCT, and their care practices, effectively reshaping the home into a space capable of supporting high-acuity care.

These pumps require no adjustment. They don’t require programming by nurses, or additional tubing; less nursing time is needed – their flow rate is predetermined. Some pharmacies can even prepare them in advance, for example, hydromorphone. We must do all we can to avoid leaving a dying patient to suffer at home.^55^

Called a “baby bottle” by the participants because of its shape, this pharmacological device^54^ is sometimes used in palliative care^56^ and was a central device in the enablement of death at home for the COVID-IHCT.^53^ The participants explained the value of the pumps within the context of multiple patients dying at home at the same time, lack of other pumps (e.g., patient self-managed analgesia), inadequate home care staff to prepare syringes, limited family assistance in administering injections, and pharmacies lacking the time to prepare refilled syringes. These accounts were corroborated on the HCPs’ website Palli-Science.^55^ However, for all its convenience, the pump could not replace real social interaction at the heart of a dignified end-of-life experience.^32^ New care technologies transformed the conventional care environment, blurring the lines between the public and private spheres, and raised concerns about who truly benefits from these innovations.^3^

#### The “tackle box”: Pharmacy at your fingertips

Additionally, each team member had access to an emergency medication kit, prepared by the IHSSC, known as the “tackle box,” eliminating the need to wait for prescriptions from a private pharmacy in the community. This immediate access to essential medications empowered the IHCT to respond swiftly to patients’ needs, similar to the access to resources available in hospital settings. Each team member had access to the kit, including medications often used in emergency and end-of-life care. One participant explains the benefits of the kit:

It was like a tackle box. . . . We were wondering what we needed to do hospitalization at home, because that’s what the IHCT is all about. So, what does the hospital have to treat its patients? We need it too, and we don’t have time to wait for a prescription from the pharmacy, which will be delivered in four hours. No, no, no. I open my briefcase and there it is. (Director continuum of care – IHSSC A, M1)

According to the participants, this medication kit encouraged a proactive and reactive approach to care, empowering the COVID-IHCT to be more efficient. It gave nurses and doctors greater professional autonomy, enabling them to intervene directly in patients’ homes.

#### Control by the hospital pharmacy

The participants highlighted the acute logistical challenges associated with end-of-life medication, particularly the management of morphine and the preparation of the “baby bottle,” which were addressed through the centralization of pharmacy services. By shifting responsibility from private community pharmacies to the hospital pharmacy, delays in medication administration were mitigated.

At first, we dealt with community pharmacies for baby bottles, but delivery took far too long. The patient had time to die in pain. We stopped that. We asked the [hospital] pharmacy to supply the bottles. So, we carried bottles with us at all times. That way, as soon as we saw signs of respiratory distress and then the level of care allowed us to start a level of comfort care, we started comfort care quickly. (Nurse – COVID-IHCT, HCP 10)

This transition was characterized by the centralization of services at the IHSSC, prioritization of IHCT needs, proactive planning with advance bottle preparation, and close coordination with the team for delivery. This initiative not only reduced waiting times but also ensured the immediate availability of necessary supplies. Overall, medication management proved to be a cornerstone of the team’s intervention to provide end-of-life care at home.

### Reconfiguring the RCF space

#### Use of virtual care

During the initial COVID wave, family caregiver visits were largely restricted in RCFs, except for compassionate visits for imminent death. When a family member was able to attend, their presence providing comfort care was impactful despite the COVID-19 limitations:

The daughter had come to the apartment. Of course, when you’re a child, you’re a little younger, so there’s less risk. So, she often came to care for her mother, who had COVID, at home in the last few days. It was a case where we didn’t have a hospital bed, and the daughter tried to place lots of pillows to position her properly, because it was a bed. . . . it was an apartment. It was quality life care in people’s own homes, and people were very grateful. (Physician – COVID-IHCT, HCP 12)

However, these visits were difficult to plan, as either the relatives were unable or unwilling to visit for fear of catching COVID-19, or the COVID-IHCT team arrived at the last minute or too late, and the patient had already died. Participants noted the challenge of maintaining communication between patients and their loved ones among these restrictions. In some instances, despite the barriers of masks, gowns, visors, and gloves, the COVID-IHCT managed to foster moments of intimacy between patients and their families through phone calls or FaceTime. Alternatively, the physician or nurse would maintain connections by contacting families, sharing updates, and offering reassurance. Such connections were deeply valued:

I think what touched me the most was when I called the families every day to tell them that their loved one was not doing so well and that, unfortunately, they were approaching the end of their life. How grateful the families were that we were there, and how grateful they were that they could stay in their homes and do their own thing. (Physician – COVID-IHCT, HCP 12)

The participants described how they would comfort families by assuring them that their loved ones had died peacefully with the COVID respiratory distress protocol, despite the limited ability of staff to directly observe the exact circumstances of the patients’ deaths. The idea of dying alone was viewed unfavorably, and trying to prevent such situations created stress among staff.^5^ The participants expressed the desire to connect the patient “to home,” that is, to their loved ones.^1^ Toward the end of the first wave, there was a slight relaxation of visitation restrictions due to the advocacy of people recognizing the consequences of patients dying alone.

#### Setting up a “base camp.”

COVID-IHCT managers set up an operational center. The “COVID-IHCT base camp” was a centralized location in the heart of the area, at the health center, created to coordinate the activities of the two IHCT teams dedicated to the care of patients with COVID-19. This space served as a daily meeting point from Monday to Friday, and brought together staff caring for patients with COVID-19. The base camp was also the venue for numerous meetings, used for updates and continuing education sessions on COVID-19 and palliative care. In addition, this centralized location functioned as a coordination space to prioritize services, resolve conflicts, and discuss ethical dilemmas encountered by the team. The assistant head nurse updated a list of patients to be seen for the day. Kits containing medication and equipment were prepared at the hospital and stored at this location, providing an essential “operational base” to ensure “effective” management of patients with COVID-19 at home.

The nurse manager in charge of centralizing and coordinating all activities for the COVID-IHCT team, with the head physician, covered each territory. For the duration of the crisis, the IHCT team supplemented the home care team already in place (visiting patients with conditions other than COVID-19) and the palliative home care team (visiting patients receiving palliative care for conditions other than COVID-19).

#### Setting up transitional places

The participants highlighted the use of various zones in RCFs, designed to reduce the risk of transmission. Unlike in a hospital or long-term care unit, it was impossible to create red zones for patients with COVID-19 in RCFs. Each resident had their own furniture and personal belongings in a rented room. The only “red” or contaminated area was the room of the resident with COVID-19, unless the situation was so devastating that all the residents on the floor became infected. The COVID-IHCT had to follow a complex procedure each time they entered or left a room, which involved multiple steps such as washing hands, removing and disinfecting protective gear, and transferring items to new bags.

Some participants mentioned contacting colleagues working in other regions of Québec where the IHCT had an alternate care site for patients with COVID-19. Patients with COVID-19 from RCFs without on-site nursing resources were transferred directly to these sites, thus pooling all the necessary human and material resources. This saved travel time for nurses, allowed the same protective equipment to be used in the red zone, and enabled one nurse to care for several patients simultaneously, thus improving care efficiency. The lack of access to an alternative care site was seen as a major shortcoming: “All the problems that ensued could have been solved by this to a large extent” (Physician – COVID-IHCT, HCP 9).

Many of the participants wanted access to an alternate care site to offer all basic care with a palliative care approach, socialization, oral care, hydration, hygiene, and repositioning. When the alternate care site opened in late May, the patients with COVID-19 with cognitive impairment and wandering problems, and those with care Level 3 or 4, could be transferred there instead of remaining in an RCF. However, it arrived “too late” as the intense 3 weeks of “hecatomb” with multiple outbreaks were over. This site functioned as a “transition bubble,” a temporary care place for patients too ill from COVID to be at their RCFs but too frail to be transferred to the hospital. Participants described the alternate care site as a safe place and space not only for patients but also for the caregivers, reflecting how the quality of a space for dying is determined by the individuals present, their “personhood” and how it is perceived and honored.^1^

## Discussion

The findings in this paper resonate with Travaglia and Robertson’s^19^ work on COVID-19 necropolitical spaces. The COVID-IHCT transformed RCFs into a necropolitical space. And these spaces, established by government agencies, function under transitional and uncertain conditions. They tacitly acknowledge the shortcomings of existing systems and the inadequacy of surge capacity to address real public health crises.^19^ While we focused on a particular time, area, and population in Québec, this case study highlighted how the COVID-IHCT facilitated home-based end-of-life care by redefining RCFs as extensions of institutional care.^3^

This case study contributes to a better understanding of the ambiguities and complexities of dying at home during a pandemic, and prompts reflection on the process of the institutionalization of an aging population.^3^ The COVID-IHCT intervened in a specific type of home, RCFs, a unique intersection of public and private spheres within the therapeutic landscape.^5,7^ These living places are private companies, and employ their own staff to provide basic care. Under Québec’s 2015 Health Reform,^57^ each health center assumes population responsibility for its designated area and is charged with monitoring, supporting, and overseeing the quality of services provided in those private residential facilities. However, they do not have any decision-making power with regard to the provision of care.

In March 2020, when the Québec government declared a state of public health emergency under the Public Health Act,^58^ IHSSCs could intervene in private RCFs and faced fewer bureaucratic barriers, which enabled the creation of a COVID-IHCT.^52^ The provision and administration of COVID-IHCT services in RCFs operated under the authority granted by the Public Health Act. The findings of this case study highlighted the creativity of the IHCT team while navigating the crisis, but also revealed shortcomings in the care of individuals suffering the loss of their autonomy. While Travaglia and Robertson^19^ demonstrate that decision-making processes, protocols, and criteria used in DNR orders are imbued with rationality, we also showed that the pump itself exhibits agency, which influences the decision to keep the person in the RCF.^53^ The privatization of care in Québec’s RCFs reflects broader shifts in health and social services networks driven by governmental cost reductions in response to demographic aging.^59^ These reforms have altered the roles of individuals and families and weakened collective support structures.^60,61^ Globally, neoliberal economic principles have exacerbated this trend, emphasizing individual responsibility and reliance on private resources for aging care provision.^62^

The study does not incorporate the perspectives of other key actors in the care continuum, such as patients, their families, and non-licensed workers. In addition, the reliance on virtual data collection due to health restrictions was a limitation, as in-person observations would have provided valuable insights. Furthermore, the analysis does not address intercultural complexities related to end-of-life care. Future research should broaden the lens to include the perspectives of patients, families, and non-licensed care workers, as well as explore the role of cultural and gender dynamics in shaping care practices and the transformation of RCFs during crises. By doing so, scholars and policymakers can better understand how to design aged care systems that are both humane and resilient in the face of future public health emergencies.

## Conclusion

By enabling home-based end-of-life care within RCFs, the COVID-IHCT demonstrated both the potential for flexible, compassionate interventions and the limitations of existing healthcare infrastructures, particularly under conditions of crisis. From a policy perspective, this study highlights the importance of integrating crisis preparedness and adaptive care strategies into aged care systems. Policies that support interdisciplinary teams, empower care providers, and facilitate flexible care delivery across private and public spheres could enhance resilience and ensure dignified end-of-life care.

## Supplemental Material

sj-docx-1-pcr-10.1177_26323524251407703 – Supplemental material for The COVID-19 experience and the necropolitical space of end-of-life care in residential care facilities in Quebec: A case study Supplemental material, sj-docx-1-pcr-10.1177_26323524251407703 for The COVID-19 experience and the necropolitical space of end-of-life care in residential care facilities in Quebec: A case study by Andréanne Robitaille, Johanne Collin and Pierre-Marie David in Palliative Care and Social Practice
