# Supplementary material for: The COVID-19 experience and the necropolitical space of end-of-life care in residential care facilities in Quebec: A case study
Source: Palliat Care Soc Pract. 2026 Feb 13;20:26323524251407703. doi: 10.1177/26323524251407703 (PMC12905072; doi:10.1177/26323524251407703)
Supplement: sj-docx-1-pcr-10.1177_26323524251407703 – Supplemental material for The COVID-19 experience and the necropolitical space of end-of-life care in residential care facilities in Quebec: A case study [file sj-docx-1-pcr-10.1177_26323524251407703.docx]

Completed COREQ (Consolidated Criteria for Reporting Qualitative Research) Checklist for Interviews: Manuscript Title – 'The COVID-19 Experience and the Necropolitical Space of End-of-Life Care in Quebec Residential Care Facilities: A Case Study'

| **Item No.** | **Item** | **Guide Questions** | **Manuscript Response** |
| --- | --- | --- | --- |
| 1 | Interviewer/facilitator | Which author/s conducted the interview or focus group? | AR |
| 2 | Credentials | What were the researcher’s credentials? e.g., PhD, MD | RN |
| 3 | Occupation | What was their occupation at the time of the study? | PhD student |
| 4 | Gender | Was the researcher male or female? | Female |
| 5 | Experience and training | What experience or training did the researcher have? | PhD student in social sciences |
| 6 | Relationship established | Was a relationship established prior to study commencement? | No |
| 7 | Participant knowledge of the interviewer | What did the participants know about the researcher? e.g., personal goals, reasons for doing the research | Yes |
| 8 | Interviewer characteristics | What characteristics were reported about the interviewer/facilitator? e.g., bias, assumptions, reasons and interests in the research topic | Yes |
| 9 | Methodological orientation and theory | What methodological orientation was stated to underpin the study? e.g., grounded theory, discourse analysis, ethnography, phenomenology, content analysis | Constructive case study (Merriam) |
| 10 | Sampling | How were participants selected? e.g., purposive, convenience, consecutive, snowball | Purposive |
| 11 | Method of approach | How were participants approached? e.g., face-to-face, telephone, mail, email | email |
| 12 | Sample size | How many participants were in the study? | 30 |
| 13 | Non-participation | How many people refused to participate or dropped out? Reasons? | 2 HCPs (during the second wave of COVID-19) |
| 14 | Setting of data collection | Where was the data collected? e.g., home, clinic, workplace | Virtual |
| 15 | Presence of non-participants | Was anyone else present besides the participants and researchers? | No |
| 16 | Description of sample | What are the important characteristics of the sample? e.g., demographic data, date | Yes |
| 17 | Interview guide | Were questions, prompts, guides provided by the authors? Was it pilot tested? | No |
| 18 | Repeat interviews | Were repeat interviews carried out? If yes, how many? | No |
| 19 | Audio/visual recording | Did the research use audio or visual recording to collect the data? | Yes |
| 20 | Field notes | Were field notes made during and/or after the interview or focus group? | Yes |
| 21 | Duration | What was the duration of the interviews or focus group? | Yes |
| 22 | Data saturation | Was data saturation discussed? | Yes |
| 23 | Transcripts returned | Were transcripts returned to participants for comment and/or correction? | No |
| 24 | Number of data coders | How many data coders coded the data? | AR, JC, PMD |
| 25 | Description of the coding tree | Did authors provide a description of the coding tree? | No |
| 26 | Derivation of themes | Were themes identified in advance or derived from the data? | Derived from the data |
| 27 | Software | What software, if applicable, was used to manage the data? | Yes |
| 28 | Participant checking | Did participants provide feedback on the findings? | Yes |
| 29 | Quotations presented | Were participant quotations presented to illustrate the themes/findings? Was each quotation identified? e.g., participant number | Yes |
| 30 | Data and findings consistent | Was there consistency between the data presented and the findings? | Yes |
| 31 | Clarity of major themes | Were major themes clearly presented in the findings? | Yes |
